# Psychometric Properties of the Hospital Anxiety and Depression Scale in Individuals With Chronic Obstructive Pulmonary Disease: Protocol for a Systematic Review

**DOI:** 10.2196/37854

**Published:** 2022-09-22

**Authors:** Aleksandra Nikolovski, Lara Gamgoum, Arshpreet Deol, Shea Quilichini, Ethan Kazemir, Jonathan Rhodenizer, Ana Oliveira, Dina Brooks, Sanaa Alsubheen

**Affiliations:** 1 School of Rehabilitation Science McMaster University Hamilton, ON Canada; 2 West Park Healthcare Centre Toronto, ON Canada; 3 Lab 3R Respiratory Research and Rehabilitation Laboratory School of Health Sciences University of Aveiro Aveiro Portugal; 4 Department of Physical Therapy and Rehabilitation Science University of Toronto Toronto, ON Canada

**Keywords:** COPD, HADS, reliability, responsiveness, validity, anxiety, depression, mental health, pulmonary, systematic review, protocol, mortality, functioning, quality of life, tool, symptoms, database

## Abstract

**Background:**

In individuals with chronic obstructive pulmonary disease (COPD), anxiety and depression contribute to increased mortality and exacerbations, decreased physical functioning, and deteriorated health-related quality of life. The Hospital Anxiety and Depression Scale (HADS) is a patient-reported tool developed to measure symptoms of anxiety and depression in clinical settings. The HADS has been frequently used with individuals with COPD; however, its measurement properties lack critical appraisal in this population.

**Objective:**

This review aims to summarize and critically appraise the validity, reliability, and responsiveness of the HADS in individuals with COPD.

**Methods:**

Five electronic databases (MEDLINE, Embase, Scopus, PsychINFO, and Web of Science) will be systematically searched. Articles will be included if they assessed the measurement properties of the HADS in COPD; were published in a peer-reviewed journal; and were written in English. The COSMIN (Consensus-based Standards for the Selection of Health Measurement Instruments) guidelines will be used to assess the methodological quality and level of evidence in the selected studies.

**Results:**

To date, 12 articles met the inclusion criteria and will be included in the systematic review. The results of the psychometric properties of HADS will be qualitatively summarized and compared against the criteria for good measurement properties. The overall quality of evidence will be graded using the modified Grading of Recommendations, Assessment, Development and Evaluation approach. We expect to complete the systematic review by December 2022.

**Conclusions:**

This systematic review will be the first to evaluate the psychometric properties of the HADS in individuals with COPD. Given the negative impact of anxiety and depression on physical functioning and health-related quality of life, this systematic review provides an opportunity to use the HADS as a validated measurement tool for the assessment and treatment of anxiety and depression in individuals with COPD.

**Trial Registration:**

PROSPERO CRD42022302064; https://www.crd.york.ac.uk/prospero/display_record.php?RecordID=302064

**International Registered Report Identifier (IRRID):**

PRR1-10.2196/37854

## Introduction

Chronic obstructive pulmonary disease (COPD) is a common treatable but incurable lung disease that is characterized by persistent airflow limitation due to the significant exposure to noxious particles or gases resulting in a chronic inflammatory airway response [1]. COPD is strongly linked to a history of smoking and is associated with advanced age [2]. Globally, the World Health Organization estimates that COPD will be the third-leading cause of death in the world by 2030 [3].

Anxiety and depression are two of the most common and least treated comorbidities in individuals with COPD [4] with an estimated prevalence of 10% to 86% [4,5]. In general medical patients, anxiety disorders involve excessive feelings of fear, tension, and worry; changes in behavior such as avoidance or panic attacks; and physical manifestations such as tachycardia and hyperhidrosis [6]. Depressive disorders include emotions of sadness, loss of energy, anhedonia, and feelings of hopelessness, and can cause cognitive and somatic symptoms such as decreased appetite, fatigue, trouble sleeping, and difficulty concentrating [7].

Women and older adults (aged >65 years) with COPD are at an increased risk of developing both anxiety and depression [8,9]. Furthermore, individuals with COPD are 85% more likely to develop anxiety disorders than their healthy age-sex matched controls [10] and patients with other chronic diseases [11]. Anxiety is also reported to cause dyspnea at earlier stages of COPD [12,13], and both disorders are associated with decreased physical functioning and deteriorated health-related quality of life [14,15]. The assessment and timely interventions for these symptoms is essential, as anxiety and depression are associated with increased mortality, COPD exacerbations, hospitalizations [16,17], and medical costs [18].

The Hospital Anxiety and Depression Scale (HADS) is a self-assessment tool developed to measure symptoms of anxiety and depression in clinical settings [19], and is used for individuals with COPD [20,21]. The scale consists of 14 items, with seven items assessing anxiety and seven items assessing depression. Items are rated on a 4-point Likert scale: zero (not present) to 3 (severe symptoms). Scores range from 0 to 21 for each subscale and can be summed to give a total anxiety-depression score with a maximum of 42 points. Higher scores indicate an increasing severity of symptoms, with a cutoff score of 8 indicating high symptoms of depression and anxiety [22].

Several systematic reviews examined the criterion validity of the HADS and reported a wide range of specificity (54%-95%) and sensitivity (44%-100%) values of the HADS in patients with mental disorders [23], impaired physical health [24], cancer [25], cardiac disease [26], multiple sclerosis [27], and COPD [28]. However, the methodological quality and level of evidence for criterion validity [28] and other measurement properties has not been evaluated and critically appraised for individuals with COPD.

We aim to evaluate and critically appraise the validity, reliability, and responsiveness of the HADS in individuals with COPD. Such an evaluation will provide robust measurement information for clinicians and researchers involved in respiratory care and guide the clinical assessment and management of anxiety and depression in individuals with COPD.

## Methods

The systematic review will be conducted according to the COSMIN (Consensus-based Standards for the Selection of Health Measurement Instruments) guidelines for systematic reviews [29], and the results will be reported following the PRISMA (Preferred Reporting Items for Systematic Reviews and Meta-Analyses) guidelines [30]. The protocol registration number on PROSPERO is CRD42022302064.

### Eligibility Criteria

Studies will be included if they assessed the psychometric properties (reliability, validity, responsiveness) of the HADS in adults 18 years and older who are diagnosed with COPD, are published in a peer-reviewed journal, and are written in the English language.

Studies will be excluded if they used the HADS as an outcome measure in an interventional study and did not assess its psychometric properties; included people younger than 18 years; assessed the psychometric properties of the HADS in patients with chronic disease other than COPD; included individuals with COPD during/immediately after acute exacerbations (within 4 weeks); and are systematic reviews, conference publications, abstracts, posters, editorials, and commentaries of the HADS.

### Search Methods for Identification of Studies

Two authors (AD and SQ) will independently conduct a systematic electronic search in five digital databases (MEDLINE, Embase, Scopus, PsychINFO, and Web of Science) from inception until March 2022 using the following keywords: reliability OR validity OR responsiveness OR psychometric properties OR measurement properties AND Hospital Anxiety and Depression Scale OR HADS AND Chronic Obstructive Pulmonary Disease OR COPD. In addition, the reference list of the selected studies will be manually searched to identify further relevant articles. Endnote (Clarivate) will be used to remove duplicates, and nonduplicated articles will be uploaded to Covidence for screening. An example of the search strategy in MEDLINE is reported in Multimedia Appendix 1.

### Study Selection

**Figure 1:**
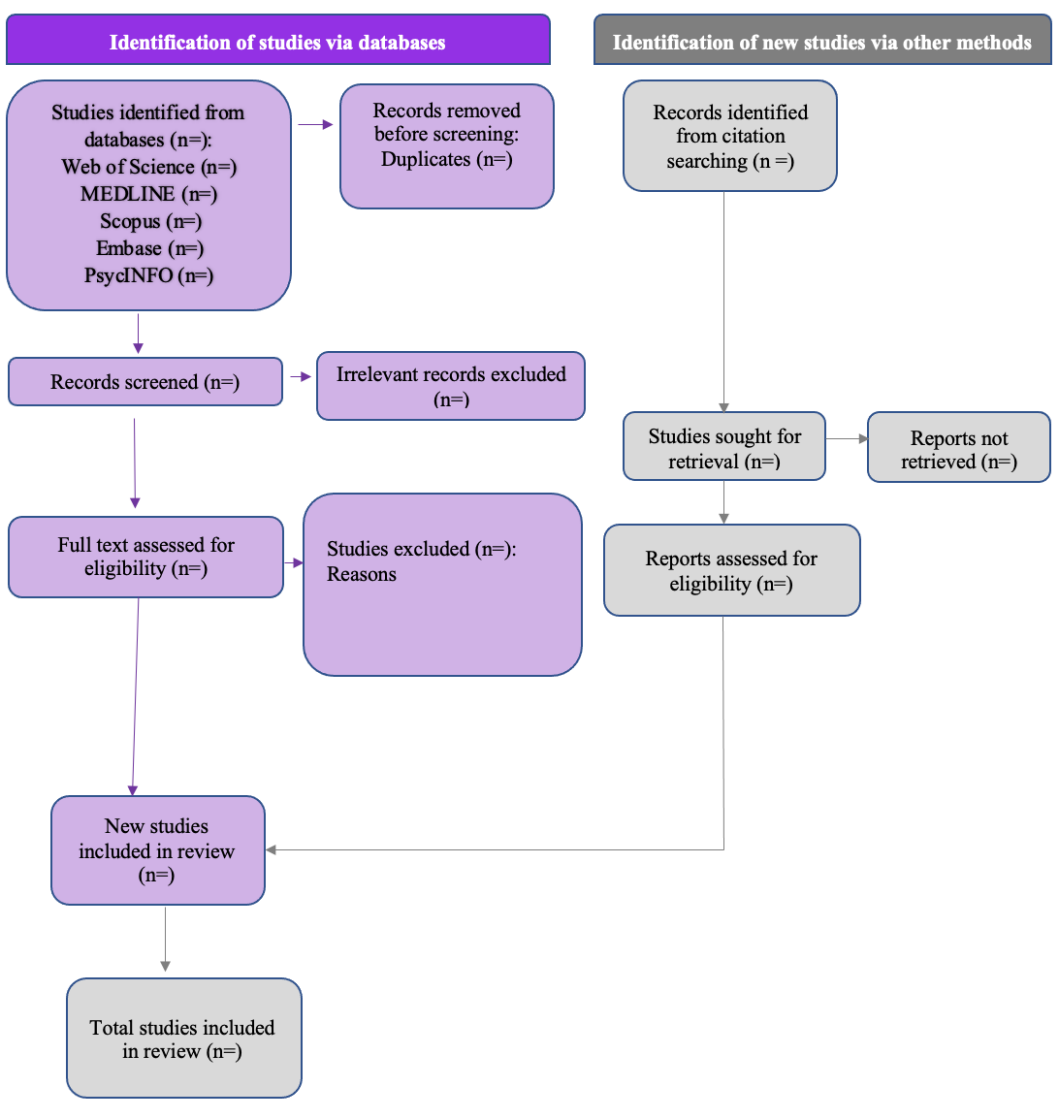
PRISMA (Preferred Reporting Items for Systematic Reviews and Meta-Analyses) flow diagram showing the proposed study selection process for the Hospital Anxiety and Depression Scale.

Two authors (AD and EK) will independently perform screening of the titles and abstracts of the identified studies. Articles that appear to meet inclusion criteria will be included at this stage. These articles will be retrieved with full text and will be screened independently by two reviewers. Disagreements between the two authors will be resolved by discussion with a senior research team member. An example of the PRISMA flow diagram showing the proposed study selection process for the HADS is presented in Figure 1.

### Data Extraction

Two authors (AN and EK) will independently carry out data extraction. The data extracted from individual studies will include the last name of the first author, year of publication, country, original language and available translations, study design, number of participants, age and sex of participants, and COPD severity. The HADS score and its psychometric properties including content, construct, criterion validity, reliability (internal consistency and measurement error), and responsiveness will also be extracted in accordance with COSMIN guidelines [29]. After completion, all data will be cross reviewed by the senior author for accuracy and completeness.

### Methodological Quality

Two authors will independently evaluate the methodological quality of each selected study on psychometric properties using the COSMIN Risk of Bias checklist [29]. The results of the psychometric properties will be qualitatively summarized for all psychometric properties and compared against the criteria for good measurement properties to rate the overall summary result of the scale as sufficient (+), insufficient (–), indeterminate (?), or inconsistent (±). A meta-analysis will be performed for the intraclass correlation coefficients if we find enough studies assessing test-retest reliability of the HADS in COPD. The overall quality of evidence will be graded as high, moderate, low, or very low using the modified Grading of Recommendations, Assessment, Development and Evaluation approach [29].

### Hypothesis Testing for Construct and Criterion Validity and Responsiveness

To interpret the results of hypothesis testing and assess the quality of evidence for construct and criterion validity and responsiveness, the review team will formulate a set of hypotheses about the expected relationships (direction and magnitude) of the HADS and other instruments and variables based on COSMIN recommendations [29]. These hypotheses will be based on the literature and the clinical experiences of the review team. The review team expect strong correlations (*r*≥0.50) between the HADS and anxiety and depression scales (similar construct), moderate correlations (0.30≤*r*<0.50) with related constructs such as positive affect, and weak or no correlations (*r*<0.30) for unrelated constructs such as demographics. An example of the expected hypotheses is summarized in Multimedia Appendix 2.

## Results

Five systematic databases will be searched for articles investigating the psychometric properties of the HADS. Currently, 12 articles met the inclusion criteria and are included in the systematic review. The results of the psychometric properties of the HADS will be qualitatively summarized and compared against the criteria for good measurement properties. The methodological quality of each selected study will be evaluated using the COSMIN Risk of Bias checklist [29]. The overall quality of evidence will be graded as high, moderate, low, or very low. We expect to complete the systematic review by December 2022.

## Discussion

Anxiety and depression can negatively impact physical functioning, health-related quality of life, and adherence to pulmonary rehabilitation programs in individuals with COPD. This systematic review provides an opportunity to use the HADS as a validated measurement tool for the assessment and treatment of anxiety and depression in individuals with COPD in the clinical setting. This systematic review presents several strengths and limitations. It will be the first systematic review to evaluate and critically appraise the psychometric properties of the HADS in individuals with COPD. Additionally, a comprehensive literature search across five databases will be conducted. The limitations of this review include the possibility of excluding articles written in languages other than English and examining other chronic respiratory disorders.

## Appendix

Multimedia Appendix 1 MEDLINE search strategy.

Multimedia Appendix 2 Hypothesis testing for criterion and construct validity and responsiveness.

## Abbreviations

COPD: chronic obstructive pulmonary disease
COSMIN: Consensus-based Standards for the Selection of Health Measurement Instruments
HADS: Hospital Anxiety and Depression Scale
PRISMA: Preferred Reporting Items for Systematic Reviews and Meta-Analyses
