# Supplementary material for: Psychometric Properties of the Hospital Anxiety and Depression Scale in Individuals With Chronic Obstructive Pulmonary Disease: Protocol for a Systematic Review
Source: JMIR Res Protoc. 2022 Sep 22;11(9):e37854. doi: 10.2196/37854 (PMC9539646; doi:10.2196/37854)
Supplement: Multimedia Appendix 1 [file resprot_v11i9e37854_app1.docx]

**Supplement 1**

*MEDLINE search strategy*

|  | **Searches** | **Strategy Utilized** | **Results** |
| --- | --- | --- | --- |
| 1 | exp Psychometrics/ | Explode key term | 83139 |
| 2 | psychometric propert*.mp. | Truncation | 27782 |
| 3 | exp “Reproducibility of Results”/ | Explode key term, quotation marks for exact phrase | 440071 |
| 4 | Psychometric*.mp. | Truncation of key term | 106603 |
| 5 | measurement propert*.mp. | Truncation of key term | 3298 |
| 6 | Reliab*.mp. | Truncation of key term | 545178 |
| 7 | Valid*.mp. | Truncation of key term | 898903 |
| 8 | Responsive*.mp. | Truncation of key term | 254717 |
| 9 | “Hospital anxiety and depression scal*”.mp. | Truncation of key term, quotation marks for exact phrase | 10477 |
| 10 | Hospital anxiety depression scal*.mp. | Truncation of key term | 524 |
| 11 | HADS.mp. |  | 6547 |
| 12 | exp Pulmonary Disease, Chronic Obstructive | Explode key term | 62069 |
| 13 | exp Lung Diseases, Obstructive | Explode key term | 225479 |
| 14 | Chronic obstructive pulmonary disease.mp. | Explode key term | 55374 |
| 15 | Chronic obstructive lung disease.mp. |  | 4405 |
| 16 | Chronic obstructive respiratory disease.mp. |  | 93 |
| 17 | COPD.mp. |  | 5305 |
| 18 | 1 OR 2 OR 3 OR 4 OR 5 OR 6 OR 7 OR 8 | BOOLEAN OR | 1829090 |
| 19 | 9 OR 10 OR 11 | BOOLEAN OR | 11823 |
| 20 | 12 OR 13 OR 14 OR 15 OR 16 OR 17 | BOOLEAN OR | 250455 |
| 21 | 18 AND 19 AND 20 | BOOLEAN AND | 76 |
