# Supplementary material for: Psychometric Properties of the Hospital Anxiety and Depression Scale in Individuals With Chronic Obstructive Pulmonary Disease: Protocol for a Systematic Review
Source: JMIR Res Protoc. 2022 Sep 22;11(9):e37854. doi: 10.2196/37854 (PMC9539646; doi:10.2196/37854)
Supplement: Multimedia Appendix 2 [file resprot_v11i9e37854_app2.docx]

**Supplement 2**

*Hypothesis Testing for criterion and construct validity and responsiveness*

| Measurement property | Expected differences and correlations (magnitude and direction) |
| --- | --- |
| **Criterion validity** | |
| MINI (Gold standard) | Strong and positive (r ≥ 0.70 or AUC ≥ 0.70) |
| **Convergent validity and responsiveness** | |
| Similar constructs: Anxiety and depression scales  Related to anxiety and depression constructs:  - Negative emotions such as empty, worried, anxious, tense, sad, panic  - Personality traits such as shy, self-enclosed, restless  - Having other mental health problems  - Measures of social isolation and loneliness  - Measures of psychology and psychiatry unless include anxiety and depression assessment  - Health-related behaviors (alcohol and drug use, quality of sleep)  - Positive emotions such as happiness  - Personality traits such attractive, open, agreeable, relaxed  - Measures of life satisfaction and quality of life | Strong and positive (r ≥ 0.50)  Moderate (0.30 ≤ r < 0.50), positive  Moderate (0.30 ≤ r < 0.50), negative |
| **Divergent validity, unrelated to anxiety and depression construct** | |
| - Physical health status  - Demographics such as age, gender, education level | Weak (r < 0.30) or no correlation |
| **Discriminative validity and responsiveness** |  |
| - Demographics: sex, gender, age, race, education level  - COPD and other CRD  - Individuals with anxiety or depression versus without | Non-significant differences between groups  Significant differences between groups |
| **Responsiveness, before-after test** | Effect size ≥ 0.2  MCID ≥ 1.5 |

AUC: area under the curve, COPD: chronic obstructive pulmonary disease, CRD: chronic respiratory disease, MCID: minimal clinical important difference, MINI: Mini-International Neuropsychiatric Interview.
